# The Genes Encoding Small Leucine-Rich Proteoglycans Undergo Differential Expression Alterations in Colorectal Cancer, Depending on Tumor Location

**DOI:** 10.3390/cells10082002

**Published:** 2021-08-06

**Authors:** Maria Pilar Solis-Hernandez, Carla Martín, Beatriz García, Natalia Pérez-López, Yolanda García-Mesa, Sara González-Fernández, Olivia García-Suárez, Jesús Merayo, Iván Fernández-Vega, Luis M. Quirós

**Affiliations:** 1 Department of Medical Oncology, Hospital Universitario Central de Asturias, Av. Roma, s/n, 33011 Oviedo, Spain; mpsolishernandez@gmail.com; 2 Department of Functional Biology, University of Oviedo, Av. Julián Clavería, 6, 33006 Oviedo, Spain; cmartincueto@gmail.com (C.M.); garciafernandezbeatriz@gmail.com (B.G.); natalia_perlop@hotmail.com (N.P.-L.); saragonzalezfernandez7@gmail.com (S.G.-F.); 3 Instituto Universitario Fernández-Vega, University of Oviedo, Av. Drs Fernández Vega, 34, 33012 Oviedo, Spain; merayo@fio.as; 4 Department of Morphology and Cell Biology, University of Oviedo, Av. Julián Clavería, 6, 33006 Oviedo, Spain; yolandagm_navia@hotmail.com (Y.G.-M.); garciaolivia@uniovi.es (O.G.-S.); 5 Department of Surgery, University of Oviedo, Av. Julián Clavería, 6, 33006 Oviedo, Spain; 6 Department of Pathology, Hospital Universitario Central de Asturias, Av. Roma, s/n, 33011 Oviedo, Spain

**Keywords:** colon cancer, SLRP, proteoglycan, extracellular matrix

## Abstract

Small leucine-rich proteoglycans (SLRPs) regulate different processes and undergo significant alterations in various diseases. Colon carcinomas (CCs) are heterogeneous pathologies with important clinical and molecular differences depending on their location, which makes it interesting to analyze the alterations in SLRPs in right- and left-sided tumors (RS- and LSCCs). SLRP transcription levels were studied in 32 CCs using qPCR compared to healthy colon mucosae samples from the same patients, 20 of them from LSCCs and the remaining 12 from RSCCs. Protein expression of genes with significant differences in their transcriptions was analyzed by immunohistochemistry. The alterations observed were related to survival data. The arrangement of transcription of SLRPs was quite similar in ascending and descending colon, but RS- and LSCCs displayed different patterns of alteration, with a greater number of deregulations occurring in the latter. The analysis of protein expression also indicated changes in the location of these molecules, largely moving to the cell interior. While podocan underexpression showed a trend toward better outcomes, no differences were observed in terms of overall survival. In vitro studies using the HT29 tumor cell line suggest that deregulation of SLRPs could affect cell proliferation. SLRPs constitute new differential markers of RS- and LSCCs, showing differences dependent on the anatomical location of the tumor.

## 1. Introduction

While colon cancer (CC) CCs usually present with a similar histological appearance, they in fact constitute a complex disease, very dependent on the anatomic primary tumor location (PTL) [1]. Right-sided colorectal cancers (RSCCs) include those of the ascending colon, as well as the cecum and two-thirds of the transverse colon, while left-sided colorectal cancers (LSCCs) comprise the descending and sigmoid colon, and the distant third of the transverse colon [1]. Both cancer subtypes display notable differences which include alterations of the microbiome, of gene expression in the mucosa, chromosomal instability or hypermutation [2,3,4,5]. In addition, PTL has an important influence on clinical aspects of the disease, including diagnosis and response to chemotherapy [6].

Various genes involved in the etiology of CCs have been described, along with their possible diagnostic and therapeutic applications [7]. While the majority of the molecules identified are part of the cellular compartment, some studies have also detected the deregulation of some extracellular matrix (ECM) proteins [8].

The ECM is a complex and organized structure that provides cells with structural support and is also able to regulate cellular behavior and homeostasis [9]. The structure and composition of the ECM undergoes significant alterations during tumour development, which plays a role in the progression of cancer [9]. In colorectal tumors, the deregulation of different components of the ECM, including its main component, collagen, has been described [8,9].

Small leucine-rich proteoglycans (SLRPs) play an essential role in the assembly of the ECM. They appear mainly associated with collagens, carrying out regulatory functions in relation to growth, organization and protection from the cleavage of collagen fibrils [10]. SLRPs are also critical in the regulation of processes such as migration, proliferation, differentiation and apoptosis [11]. In various pathologies, including cancer and inflammation, it has been possible to determine the existence of an abnormal expression of SLRPs, which leads to alteration of tissue functions [10].

SLRPs are a group of mainly extracellular molecules. In humans there are 17 genes that code for SLRPs. Located on seven chromosomes, they are grouped into five classes: Classes I–III are constituted by canonical and IV and V by non-canonical genes [12]. Class I includes biglycan (BGN), decorin (DCN), asporin (ASPN) and ECM2; class II comprises fibromodulin (FMOD), lumican (LUM), PRELP, keratocan (KERA) and osteoadherin (OMD); class III includes epiphycan (EPYC), opticin (OPTC) and osteoglycin (OGN); class IV comprises chondroadherin (CHAD), nyctalopin (NYX) and Tsukushi (TSKU); and finally, class V is composed of podocan (PODN) and podocan-like 1 (PODNL1). Many SLRPs are proteoglycans (PGs) composed of a protein core covalently linked to glycosaminoglycan (GAG) chains. Two different species of GAGs can appear in SLRPs: Keratan sulfate and chondroitin sulfate (CS), depending on the type of core protein [12].

Aberrant alterations in SLRPs have been described in various tumors and related to the control of tumor progression [13,14,15,16], although no detailed studies have been carried out in the case of CCs. Our group has previously described alterations in heparan sulfate (HS) PGs, as well as in the genes responsible for the GAGs associated with them, which includes chains of HS but also of CS depending on the specific PG species involved [17,18]. As indicated above, some SLRPs include CS chains in their structures, as is the case with biglycan, decorin and epiphycan [12]. In our previous studies, we have described several alterations of the transcription of the genes responsible for CS synthesis that affect both polymerization and saccharidic chain modification reactions [17,18]. Interestingly, these alterations are different in LS- and RSCCs, reinforcing data indicating the importance of PTL. It is also of note that studies on HSPGs have detected aberrant expression in molecules located in the extracellular matrix in CCs, including perlecan and collagen 18A1, which supports the notion that PGs participate in the disorganization of the ECM which is associated with tumor progression [17,18].

In this article, alterations in the expression of all species of SLRPs in CCs are analyzed, taking into account PTL, in order to determine the existence of any new differences at the molecular level between LS- and RSCCs. The differential transcription of the genes involved is analyzed, as well as differences at the protein level in those that appeared altered, in an attempt to determine their levels and tissue locations. In addition, the relationship between alterations in gene expression and survival data is explored. The aim of the study is to collaborate in the understanding of CC in an attempt to identify new biomarkers and their potential future biomedical application.

## 2. Materials and Methods

### 2.1. Tissue Samples

Samples from 32 patients diagnosed with colon adenocarcinoma were provided by the Tumor Bank at the University Institute of Oncology of Asturias (IUOPA, Asturias, Spain). This study thus comprised 64 snap-frozen colon samples, 32 corresponding to tumor tissue and 32 matching non-neoplastic tissue samples from the same patients used as control. Twenty samples were from LSCCs, and the remaining 12 from RSCCs. All the samples were obtained from surgical pieces. The diagnosis of colon adenocarcinoma was made using hematoxylin-eosin-stained slides in accordance with World Health Organization (WHO) criteria and the snap-frozen tissues were stored at −80 °C prior to isolation of the RNA. All patients gave their consent, and the study was approved by the Ethics Committee on Clinical Investigation of the Hospital Universitario Central de Asturias.

### 2.2. Total RNA Isolation and cDNA Synthesis

RNA isolation was carried out using the RNeasy kit (Qiagen, Hilden, Germany), starting from tissue fragments of between 20 and 30 mg in weight, and proceeding as has been previously described [17].

For the synthesis of the cDNA, 2 μg of RNA were used, and the reactions performed using the High Capacity cDNA Transcription Kit (Applied Biosystems, Foster City, CA, USA). The procedure was carried out and the products cleaned and stored as has been previously described [17].

### 2.3. qRT-PCR Reactions

The primers located in the different exons were designed using the program Primer 3 (http://biotools.umassmed.edu/bioapps/primer3_www.cgi (accessed on 21 January 2016)), adjusting the amplicon size between 70 and 150 base pairs, using a Tm above of 77 °C whenever possible. Primer sequences are presented in Supplementary Table S1. Actin was used as a control gene to normalize the results. While some recent studies point to the existence of potentially better genes for use as controls [19,20], these same studies show divergences in the results obtained depending on the technique or type of sample analyzed. Actin has been widely used previously [17,18] and, in addition, our study incorporates an additional purification step after the cDNA is obtained, which significantly improves the efficiency and reproducibility of PCR reactions [17], probably through the elimination of inhibitory molecules. For those genes where differences between normal and tumor tissue were detected, we complemented this test by localizing the proteins by means of immunohistochemistry.

Both the reactions and the analysis of the amplification products were carried out as has been described elsewhere [17]. The amplification efficiencies of each gene were calculated using the LinRegPCR program as previously described [17]. Normalization of expression values was carried out using actin as a control gene. Statistical analysis of the data was carried out using the Mann–Whitney U test to compare the mean values between left-sided and right-sided colon samples, and a non-parametric Wilcoxon matched-pair test to compare the values between healthy and tumor tissue of the same patients, as described in a previous work [17].

### 2.4. Immunohistochemistry

To perform the immunohistochemistry, paraffin embedded tissue sections were used. First, sections were made clear by treatment with xylene, after which the paraffin was removed using decreasing concentrations of alcohol until finally ending up using only water. Next, the samples were rinsed in phosphate buffered saline (PBS) containing 1% Tween-20, heated in high pH Envision FLEX target retrieval solution at 65 °C for 20 min and, after that, incubated in the same solution for 20 min at room temperature. After blocking of endogenous peroxidase activity and non-specific binding (3% H_2_O_2_, 33% fetal calf serum), the sections were incubated overnight at 4 °C with primary antibodies.

The following antibodies were used in this study: Monoclonal mouse anti-biglycan (dilution 1:50) and rabbit polyclonal anti-PRELP (dilution 1:50), from abcam (Cambridge, UK). Rabbit anti-chondroadherin (dilution 1:100) and rabbit anti-podocan (dilution 1:50) polyclonal antibodies, from Thermo Scientific (Waltham, MA, USA). Rabbit anti-osteoglycin (dilution 1:50), from USBiological Life Sciences (Salem, MA, USA). Next, the following secondary antibodies were used at a 1:100 dilution: Anti-mouse (sc-2020) and anti-rabbit (sc-2004), from Santa Cruz Biotechnology (Santa Cruz, CA, USA).

As a chromogen, 3-3 ‘diaminobenzidine was used. Finally, samples were counterstained with hematoxylin, dehydrated and mounted in Entellan^®^ (Merck, Germany). The sections were studied and photographed (20× objective) under a microscope light (Nikon-Eclipse 80i) (Nikon Corporation, Tokyo).

The quantification of the marking for the subsequent statistical analysis was carried out by comparing the mean intensity of immunostaining of 25 images of tumor tissue and another 25 of healthy tissue using the ImageJ analysis software. [21].

### 2.5. Survival Analysis

The survival analysis was performed using the Kaplan–Meier method and the survival curves were compared with the log-rank test. Overall survival was defined as the time from CC diagnosis to death. Relapse-free survival (RFS) was defined as the time from an intervention with curative intent to the first sign of new tumor activity. Progression-free survival (PFS) was defined as the time from non-curative CC detection to the first evidence of tumor growth on the basis of RECIST (Response Evaluation Criteria in Solid Tumors). Patients with an RFS of 6 months or lower were included in the PFS analysis as in clinical practice.

### 2.6. Cell Culture

The human colon adenocarcinoma cell line HT29 was grown in DMEM (Dulbecco’s modified Eagle’s medium) (GibcoBRL, Eragny, France) supplemented with 10% (*w*/*v*) fetal bovine serum (GibcoBRL) and with one of: Penicillin G/streptomycin/Amphotericin B (10,000 IU/mL, 10,000 μg/mL, 25 μg/mL) (GibcoBRL, Grand Island, NY, USA). Cultures were incubated in 25 cm^2^ tissue culture flasks (Nunc, Roskilde, Denmark) at 37 °C in a 5% (*v*/*v*) CO^2^ atmosphere.

### 2.7. Cell Migration Assay

Cell migration assays were performed using 24-well plates as previously described [22]. The wells were coated with type I collagen (Corning, Glendale, AZ, USA) at a concentration of 5 µg/cm^2^ following the manufacturer’s instructions. Subsequently, to obtain mixed wells including both collagen + SLRP, either BGN, OGN or PRELP (Sino Biological, Wayne, PA, USA) was added to different wells at a concentration of 1 μg/cm^2^, and the plates were incubated for 1 h at ambient temperature. The remaining solution was then removed and 2 washes were carried out with PBS. Finally, the plates were allowed to dry and were stored at 4 °C until later use.

### 2.8. Cell Proliferation Assay

HT29 cells were seeded onto 96-well plates (Fisher), at a density of 2500 cells/well, that had been previously coated with type I collagen (control) or a mixture of collagen and SLRP as indicated in the previous section. The effect of the different SLRPs on cell proliferation was evaluated through a colorimetric assay using MTT (Promega) following the manufacturer’s instructions. In brief, 15 µL of the MTT solution was added to each well and incubated for 4 h at 37 °C. Then, 100 μL of the lysis solution was added, and the mixture was incubated at 4 °C overnight. The absorbance at 570 nm in each set of samples was measured using a 96-well microtiter plate reader Biotek Powe Wave XS (Biotek, Winooski, VT, USA). Five replications were made for each of the treatments.

## 3. Results

### 3.1. Analysis of Differential Gene Expression

Of the 32 patients studied, all 12 RSCCs were male, while of the 20 LSCCs seven were female and the rest male. The ages of patients with RSCCs were between 58 and 86 years, with an average of 71.2 years, those of LSCCs were between 50 and 88 years, with an average of 65.7 years, with 83% and 50%, respectively, being over 65. All tumors were T3 (muscularis propria affected) with an average size of 5 cm (2.8–14 cm) in RSCC compared to 6 cm (2.5–27 cm) in LSCC. N0 cases (no nodal infiltration) were lower in RSCC, accounting for 33% (four cases) in contrast to 50% (10) in LSCC, although there was a greater proportion of G3 (high grade) in RSCC, 33% (four cases) vs. 5% (1). The clinical data of the patients are summarized in Table 1.

### 3.2. Differential Expression of Genes Encoding SLRPs in RSCCs

**Figure 1:**
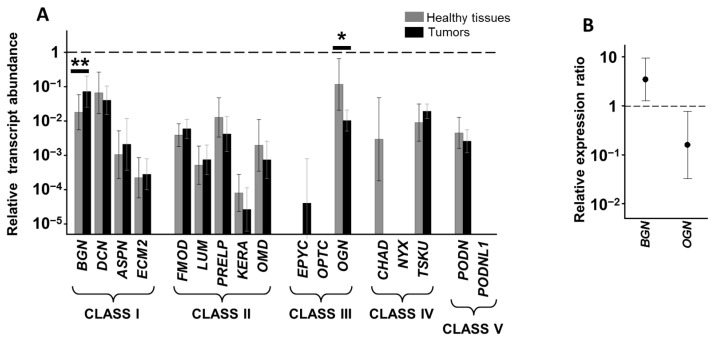
Differential transcription of SLRP genes in RSCC. (**A**) Relative transcript abundance of mRNAs for SLRPs, grouped by class. Relative abundance for healthy tissues (gray bars) and tumors (black bars) are plotted on a log scale for each gene assayed and spreads represent standard deviation. Statistically significant differences are denoted by **, and *, which indicate *p* < 0.01, and *p* < 0.05, respectively. (**B**) Relative expression ratio of genes that show statistically significant differences; the values represent the relationship between the transcription levels observed in each tumor tissue versus healthy tissue from the same patient. Values on the *Y* axis are represented on a logarithmic scale.

Of the 17 genes encoding SLRPs, transcripts of 10 (*BGN*, *DCN*, *ASPN*, *ECM2*, *FMOD*, *PRELP*, *OMD*, *OGN*, *TSKU* and *PODN*) were detected in the healthy tissues of the ascending colon of all patients analyzed in this study, while transcripts for three of the remaining genes (*LUM*, *KERA* and *CHAD*) were only quantified in around 50% of the patients, and mRNAs for the remaining four molecules (EPYC, OPTC, NYX and PODNL1) were not detected (Figure 1A).

**Figure 2:**
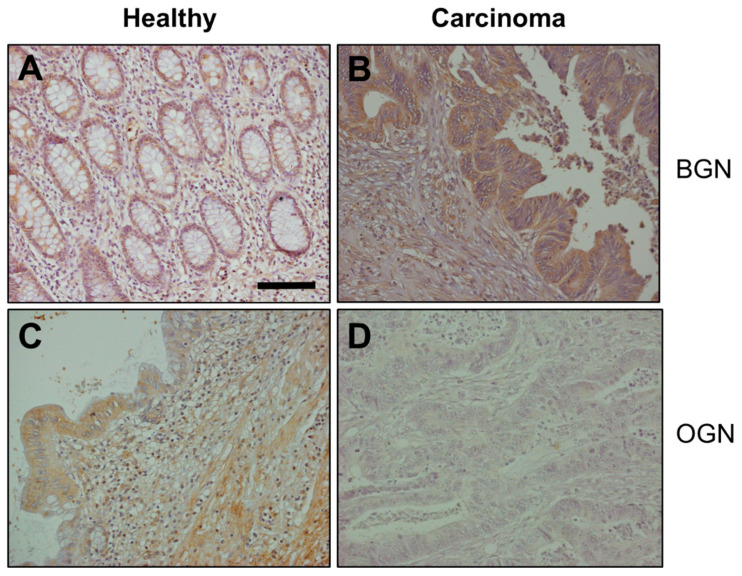
Immunolocalization of SLRPs with altered expression in RSCCs. (**A**,**B**) Histological localization of BGN expression: (**A**) Normal mucosa showing weak staining; (**B**) Tumor tissue, where BGN appears overexpressed in comparison with the normal mucosa; magnification 100×. (**C**,**D**) Histological localization of OGN expression: (**C**) Normal mucosa, showing a moderate-intense expression of OGN in the absorptive cells of the colon and in the stroma; (**D**) Tumor tissue, where expression was not detected; magnification 200×, scale bar: 100 µm.

We were able to detect mRNAs coding for all four genes related to class I SLRPs (BGN, DCN, ASPN and ECM2) in healthy tissues, although their expression levels varied notably, with BGN and DCN being the most abundant species, and the detection of transcripts for ASPN and ECM2 being around two orders of magnitude lower (Figure 1A). These same transcripts were also detected in tumor tissue, with no statistically significant differences with respect to the healthy tissue of the same patient, except in the case of BGN, where the mean of the differences between healthy and tumor tissues for each patient showed an overexpression of around 3.6-fold in CCs (*p* < 0.01, Wilcoxon test, Figure 1B). This alteration was also evident in the immunohistochemistry results, which evidenced only a weak degree of immunostaining in healthy tissue, but marked staining in tumor tissue, the difference being statistically significant (*p* < 0.01, Wilcoxon test, Figure 2A,B).

Transcripts for the five genes encoding class II SLRPs (*FMOD*, *LUM*, *PRELP*, *KERA* and *OMD*) were quantified in both healthy and tumor tissue, with no significant expression differences between tissue type (Figure 1A). The presence of transcripts for FMOD, PRELP and OMD was widespread in the patients analyzed, while expression of LUM and KERA was detected in only around 50% of cases in both healthy and tumor tissue.

When transcription of the three genes encoding class III SLRPs were analyzed, it was not possible to detect the presence of mRNAs encoding OPTC in either healthy or tumor tissue. In contrast, EPYC was not detected in healthy tissues, although low levels of expression were found in about 40% of tumor samples (Figure 1A) and, interestingly, while high levels of RNA encoding OGN were present in healthy tissue, the mean of the differences in the transcription levels between the healthy and tumor tissue samples from each patient showed that the transcription levels of this gene in tumor tissues were around 13% of those in healthy tissues (*p* < 0.05, Wilcoxon test, Figure 1B). The subexpression of OGN in RSCCs was also observed when analyzed at the protein level: Immunohistochemistry showing staining in both absorptive cells and in the stroma, with no staining detected in tumor tissue, the analysis of the differences in the images being statistically significant (*p* < 0.001, Wilcoxon test, Figure 2C,D).

In terms of the five non-canonical species of SLRPs that comprise classes IV and V, in ascending colon tissues, transcripts of TSKU and PODN were found in both tumor and healthy tissue with no statistically significant differences in their expression. Neither NYX nor PODNL1 mRNA was detected in either tissue type, while around 50% of healthy tissue samples showed variable levels of CHAD transcription, though it was totally absent in tumor tissue (Figure 1A).

### 3.3. Differential Expression of Genes Encoding SLRPs in LSCCs

**Figure 3:**
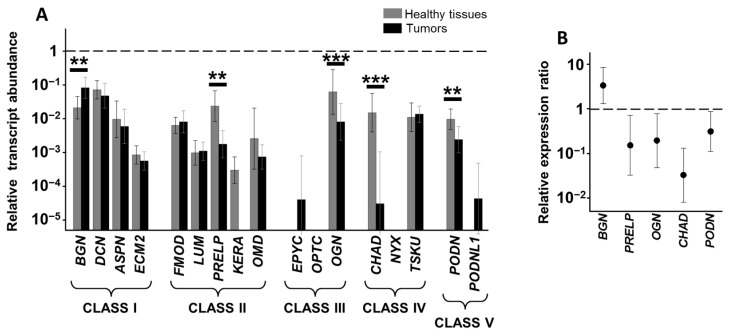
Differential transcription of SLRP genes in LSCC. (**A**) Relative transcript abundance of mRNAs for SLRPs, grouped by class. Relative abundance for healthy tissues (gray bars) and tumors (black bars) are plotted on a log scale for each gene assayed and spreads represent standard deviation. Statistically significant differences are denoted by **, and ***, which indicate *p* < 0.01, and *p* < 0.001, respectively. (**B**) Relative expression ratio of genes that show statistically significant differences; the values represent the relationship between the transcription levels observed in each tumor tissue versus healthy tissue from the same patient. Values on the *Y* axis are represented on a logarithmic scale.

The pattern of expression of the 17 species of SLRPs was very similar in healthy tissue of the descending colon to that found for the ascending colon. The comparison between the transcription levels of the healthy tissue samples from both locations using the Mann-Whitney U-test only showed significant results for ASPN (*p* < 0.01), whose levels were higher in the left colon samples. Transcripts for the same 10 (BGN, DCN, ASPN, ECM2, FMOD, PRELP, OMD, OGN, TSKU and PODN) were detected in the healthy tissues of patients, while no transcription was detected for EPYC, OPTC, NYX or PODNL1, just as in the ascending colon (Figure 3A). Similarly, transcripts of LUM, KERA and CHAD were only detected in a percentage of the patients analyzed. In terms of LUM, the percentage was similar to that of RSCC patients (50%) while for KERA it was slightly lower (around 40%) and for CHAD it was considerably higher (around 80%).

**Figure 4:**
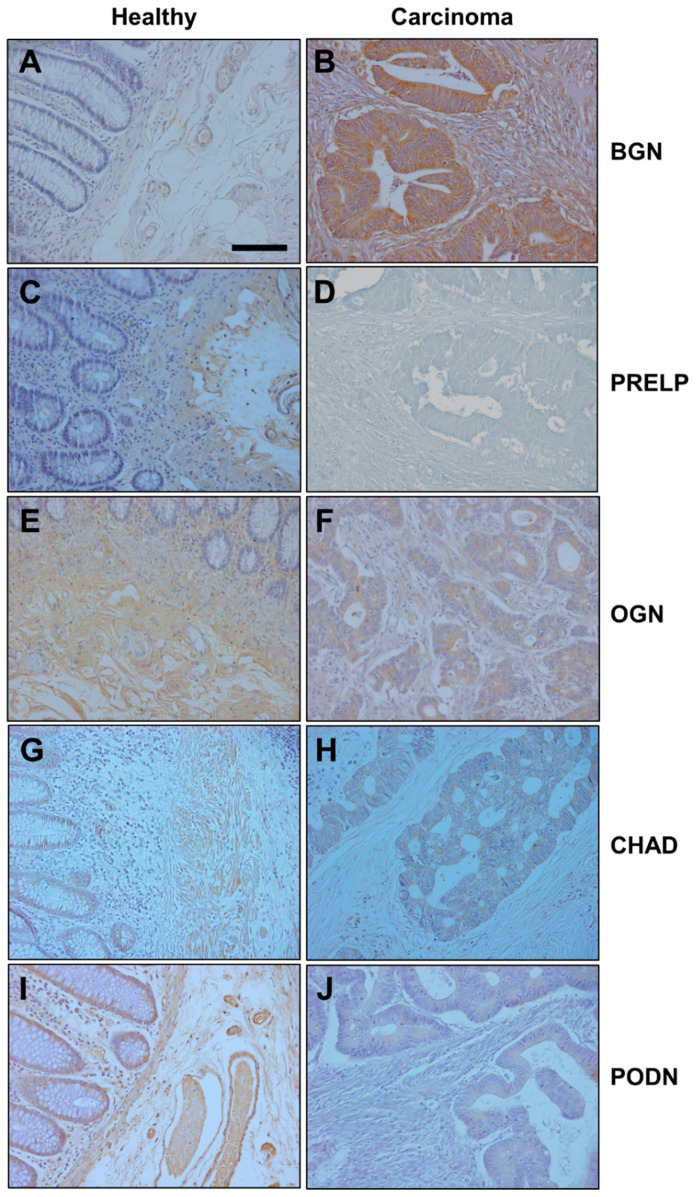
Immunolocalization of SLRPs with altered expression in LSCCs. (**A**,**B**) Histological localization of BGN expression: (**A**) Normal mucosa showing very weak staining; (**B**) Tumor tissue, showing intense expression in both the stroma and the cells. (**C**,**D**) Immunolocalization of PRELP expression: (**C**) Normal mucosa showing very weak staining; (**D**) Tumor tissue, showing intense expression in both the stroma and the cells. (**E**,**F**) Immunolocalization of OGN expression: (**E**) Healthy tissue showing moderate expression in the stroma; (**F**) Tumor tissue, in which a weak expression is observed in the tumor cells and very little in the stroma. (**G**,**H**) Immunolocalization of CHAD expression: (**G**) Normal mucosa showing weak expression in the absorptive cells and moderate expression in the stroma; (**H**) tumor sample showing weak and very focused expression in tumor cells. (**I**,**J**) Immunolocalization of PODN expression: (**I**) Control tissue in which moderate to intense expression is detected in the absorptive cells and a moderate staining in the stroma; (**J**) Neoplastic tissue showing weak staining in tumor cells. Magnification 200×, scale bar: 100 µm.

The mRNA of each of the four class I species were found in LSCC patients, and at similar levels to those observed in RSCC patients with the exception of ASPN, which was detected at significantly higher levels in healthy tissue compared to patients with RSCC (*p* < 0.01, Mann–Whitney U test). BGN, as in RSCC, was the only transcript found to be deregulated, with a fourfold overexpression similar to that observed in RSCCs (*p* < 0.01, Wilcoxon test, Figure 3B). The detection of this protein using immunohistochemistry showed very weak staining in healthy tissue, whereas expression in LSCC was intense, the differences being statistically significant (*p* < 0.001, Wilcoxon test). In addition, the labelling was detected both in the stroma and inside the tumor cells (Figure 4A,B).

In healthy tissues from the descending colon, class II SLRPs also displayed a transcription pattern that was very similar to that of ascending healthy tissue. However, in this case some differences between the two anatomical locations were detected. PRELP mRNA levels were reduced to a mean of approximately 15% in LSCC tissues compared to healthy tissues from the same patients, and this difference reached statistical significance (*p* < 0.01, Wilcoxon test, Figure 3B). This result was also evident when immunohistochemical analysis was performed, where staining was found in the stroma of healthy tissue but not in tumor tissue (*p* < 0.001, Wilcoxon test, Figure 4C,D). In addition, KERA, whose transcription, as indicated above, was detected at low levels in only 40% of the healthy tissue samples analyzed, was not detected in any LSCC tumors (Figure 3A).

The analysis of the class III SLRPs showed, once again, a pattern quite similar to that observed in the ascending colon. As in RSCC patients, it was not possible to detect RNA encoding OPTC in either tissue type, and EPYC was not found in healthy tissue and its expression in tumors was even more restricted than in RSCCs, transcripts being detectable in only 30% of patients. On the other hand, but again similar to the case with RSCCs, OGN transcripts were found at high levels in healthy tissue but experienced a 5.3-fold dysregulation in tumors compared to healthy tissues from the same patients (*p* < 0.001, Wilcoxon test, Figure 3B). This subexpression was also verified at the protein level by immunohistochemistry: Healthy tissue displayed a moderate expression of OGN in the stroma, whilst in tumoral tissues only weak staining was detected in the tumor cells and very little in the stroma (*p* < 0.001, Wilcoxon test, Figure 4E,F).

Interestingly, when the non-canonical SLRPs were analyzed, notable differences were detected with respect to the data obtained in ascending colon tissues. For class IV SLRPs, the results for TSKU and NYX were analogous to those observed in the right colon, with TSKU having comparable transcription levels and no significant differences between tumor and healthy tissue, while no transcripts for NYX were detected. However, CHAD, whose transcription was not detected at all in RSCCs, was present at notable levels in healthy tissue in LSCCs, while its expression was markedly reduced in tumor tissue and only detectable in about 50% of samples (*p* < 0.001, Wilcoxon test, Figure 3B). Immunolocalization of CHAD in normal mucosa showed weak expression in absorptive cells and moderate in the stroma, in contrast to weak focal expression in tumor cells, and the analysis of the images showed the differences to be statistically significant (*p* < 0.001, Wilcoxon test, Figure 4G,H).

Finally, regarding the non-canonical class V SLRPs, PODN transcription levels in healthy tissues of the left colon were greater than those observed in the right (*p* < 0.05, Mann–Whitney U test), but in tumor samples there was a statistically significant subexpression of around 70% compared to healthy tissue samples from the same patients (*p* < 0.01, Wilcoxon test, Figure 3A). In addition, transcription of PODNL1, whose expression was not detected in either tissue for the right colon, was found in 50% of LSCC samples, although it too was absent from healthy tissue (Figure 3A). The alteration in PODN expression was confirmed at the protein level through immunohistochemistry (*p* < 0.001, Wilcoxon test), which evidenced moderate to intense expression in absorptive cells and moderate staining in the stroma of healthy tissue, with only weak staining found in tumor cells (Figure 4I,J).

### 3.4. Relationship between Alteration in Gene Expression and Survival Data

In terms of survival, Median Relapse Free Survival (RFS) was 25.6 months (95% CI 0.00–52.145) among all patients included. After the initial complete resection of both the primary tumor and the metastases in 78.13% of the patients (25), 56% of them (14) had relapsed. Patients with RSCC had a longer RFS, 40.03 vs. 21.43 months in LSCC. While better survival results seemed to be observed when BGN was not overexpressed and when OGN was underexpressed, the results were far from statistically significant in any of the anatomical locations, so they cannot be considered an acceptable conclusion considering only the analysis of these data. When BGN was upregulated, worse outcomes were observed, with median RFS being 8.87 months when altered, and below this when BGN was not overexpressed in RSCC. The situation was similar for LSCC, where overexpression of BGN had a median RFS of 19.47 compared to 25.6 months in patients with no overexpression, although these differences were not statistically significant (*p* = 0.191). Low OGN levels were associated with longer RFS in both RSCC and LSCC, with a median of 40.03 and 27.30 months, respectively, vs. 12.63 and 19.47 months in cases with no underexpression, though once again this was not statistically significant (*p* = 0.406). In LSCC, CHAD and PRELP underexpression did not display statistical differences. Nevertheless, when downregulation of PODN was identified in LSCC it was associated with a statistically significant improvement in RFS (27.3 vs. 12.23 months, *p* 0.045, HR 0.289, 95% CI 0.086–0.975). (Detailed information is provided in Supplementary Table S2 and Figure S1.)

Progression of the disease was identified in 65.63% (21) patients, with a median Progression Free Survival (PFS) of 21.87 months (95% CI 11.51–22.89). LSCC presented a median PFS of 19.47 months vs. 17.20 months for RSCC, which was not statistically significant (*p* = 0.669, OR 1.104 95% CI 0.701–1.740). No differences in PFS were observed for BGN or OGN. However, in RSCC underexpression of BGN and OGN showed lower PFS, 3 vs. 40 months (*p* = 0.694) and 17.2 vs. 33.5 months, respectively (*p* = 0.678). Longer PFS was identified when CHAD, PRELP and PODN were downregulated in LSCC, 21 months vs. 12 months when these alterations were not identified, though none of these results were statistically significant (Supplementary Table S2 and Figure S2).

Median Overall Survival (OS) was 50 months (95% CI 33.39–66.61), and although LSCC presented better outcomes, 51.4 vs. 17.20 months in RSCC, it did not achieve statistical significance (*p* = 0.233). There was no difference in survival for OGN or BGN in any of the locations (around 50 months regardless of their level of transcription and PTL). Nor did CHAD show a difference in survival in LSCC. Better magnitudes were observed for the underexpression of PRELP (63 vs. 44 months, *p* = 0.495) and PODN (68 vs. 50 months, *p* = 0.330), though they were not statistically significant.

### 3.5. Effect of Biglycan, Osteoglycine and PRLEP on Cell Migration and Proliferation of the HT29 Cell Line

**Figure 5:**
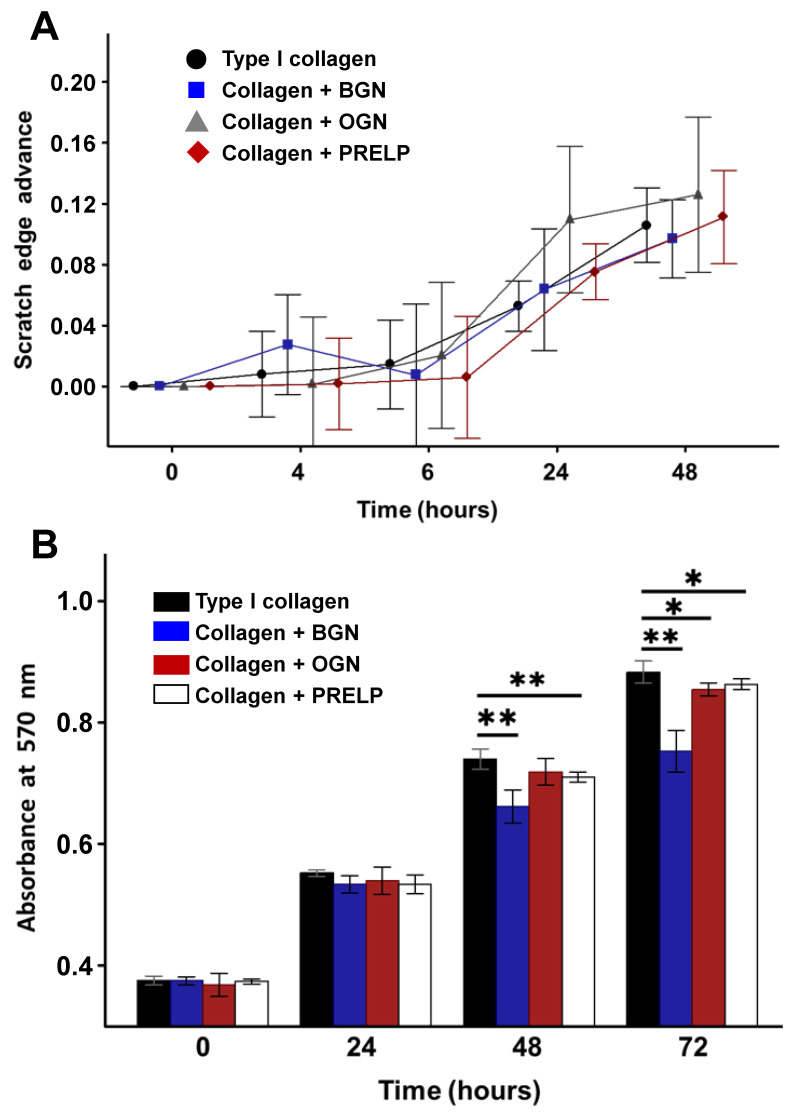
Influence of SLRPs on HT29 cell motility and proliferation. (**A**) In vitro scratch assay on plates coated with: (●) Type I collagen, (■) collagen + BGN, (▲) collagen + OGN, (♦) collagen + PRELP. (**B**) Cell Proliferation Assay on plates coated with: Type I collagen (black bars), collagen + BGN (blue bars), collagen + OGN (red bars), collagen + PRELP (white bars). Spreads represent standard deviation. Values that display significant differences are highlighted: *: *p* < 0.05, **: *p* < 0.001.

To analyze the possible effect of altering levels of SLRPs on the behavior of tumor cells, the human colon adenocarcinoma cell line HT29 was used. The cells were grown on plates previously coated with: Collagen alone (used as a control), collagen plus either BGN or OGN, the expression of both of which was altered in all CCs regardless of their location, or collagen plus PRELP, which appeared underexpressed only in LSCCs. In no case did microscopic observation of the growing cells allow the identification of any differences between the cells subjected to the different treatments (data not shown). Nor did the study of cell migration using the scratch assay technique allow any significant differences between the different experiments to be identified (Figure 5A). However, the analysis of the influence of the presence of the different SLRPs on cell proliferation did give rise to statistically significant results in all cases, the influence of BGN being particularly intense, higher than that observed for either OGN or PRELP (Figure 5B).

## 4. Discussion

An important factor in divergences displayed by CCs is the location of the primary tumor. The differences that have been described between RSCCs and LSCCs include changes in the microbiome [3,4], prognostic and clinical variations [6,23], and chromosomal and molecular alterations that affect the phenotypic expression of different biomarkers [23]. The microenvironment of a tumor is made up of cells, interstitial fluid and the ECM, and the latter experiences a great degree of disorganization related to tumor development, specifically a loss of integrity that affects collagen, the main component of the ECM, as well as the deregulation of other molecules present in the ECM [8].

The SLRPs are essential in the structure of the ECM, and several of them include CS chains [11,12]. The group consists of 17 different species, all of them extracellular with the sole exception of NYX, which is a protein anchored to the membrane through a glycosylphosphatidylinositol residue [24]. The study of the transcription of these molecules in healthy tissues of ascending and descending colon showed that the expression pattern was largely coincident in the two anatomical locations, with the most notable differences being restricted to increases in ASPN and PODN mRNA levels in the left colon. Since no transcripts were detected for NYX, all the molecules involved are located in the ECM.

The tumor transformation supposed the existence of some aberrant expressions in the SLRPs. Interestingly, the alteration patterns in RS- and LSCCs were different, with a greater number of alterations in the latter. The two most important changes in RSCCs were the overexpression of BGN and the underexpression of OGN, alterations also observed in LSCCs, along with the underexpression of PRELP, CHAD and PODN. Other differences were also detected between the two types of tumor, specifically the lack of expression of KERA and the appearance of certain levels of PODNL1, but in both cases these alterations were limited to some patients. These data thus support the theory that the expression of this molecular group shows clear differences depending on PTL, which agrees with the results previously obtained for HSPGs, including the expressions of HS and CS chains [17,18], as well as for other types of molecular markers [2,5].

Many of the genes encoding SLRPs are found forming chromosomal clusters, although no relationship was observed between those genes whose expression is altered in CCs and their chromosomal organization: The genes of BGN, PODN and CHAD are located on chromosomes X, 1 and 17, respectively, while those of OGN and PRELP are part of clusters located on chromosomes 9 and 1, respectively [25]. However, the existence of common transcription factor binding sites in many promoters of these genes has been described, suggesting the co-regulation of their expression, which could influence the pattern of alterations observed [26]. Moreover, it has also been described that the expression of SLRPs and cytokines are regulated bi-directionally through a common regulatory framework [10], CCs undergo cytokine-induced modulation, and there is increasing evidence that distinct genetic signatures may be associated with specific cytokine networks [27].

An important aspect is the fact that there seems to be a certain degree of functional overlap between the SLRPs, such that compensation mechanisms exist between them [24]. Applied to CCs, this data is interesting because in the RSCCs the two alterations observed are the overexpression of BGN accompanied by a decrease in the transcription of OGN, while in the LSCCs, the overexpression of BGN is found in conjunction with a reduction in the transcription of other genes. When the possible existence of linear correlations between these deregulations was explored, no significant result was observed between BGN and OGN in RSCCs, but a significant correlation was found between BGN and OGN, PRELP and POD in LSCCs (R = 0.69 and *p* < 0.01 in all cases), which raises the possibility that these alterations could, at least in part, be caused by compensatory mechanisms.

Correlations between mRNA and protein levels in complex biological samples often show non-linear relationships due to the existence of additional post-transcriptional mechanisms, and an example of this occurs with the expression of syndecan-1 in CCs [17,18]. However, the control of transcription usually plays an essential role in the control of gene expression and allows specific regulation depending on the cell and tissue involved [26]. The immunohistochemistry results of this study were consistent with the transcription data, suggesting its importance in the control of the expression of these molecules. However, the immunostaining highlighted an interesting fact: Decrease labelling in the ECM and its intracellular increase. The aberrant localization of SLRPs, involving intracellular localization, has been previously described in certain tumors [28]. This alteration in the location of SLRPs in CC must impact on the normal performance of their functions in relation to the assembly of the ECM, the regulation of cell-ECM interactions and the regulation of cell signalling [10]. In contrast, this change in localization might establish additional protein–protein interactions, and it has been postulated that it could even lead to changes in gene expression [28].

Only two species of SLRPs appeared to undergo deregulation regardless of the anatomic location of the tumor: BGN and OGN. BGN undergoes detectable overexpression in both RS- and LSCCs, and overexpression of this molecule has been previously described in numerous tumors, including endometrial, pancreatic, oesophageal squamous cell, gastric and prostate carcinomas [29,30,31,32,33]. Its progressive increase has also been previously described in colon tumors, when the transition from normal mucosa to adenocarcinoma takes place, although the study did not address the anatomic location of the CCs [34]. Moreover, in studies with colon cancer cell lines, BGN has been found to play an important role in proliferation, migration and invasion, along with the fact that it exerts an antiapoptotic effect [35]. In these same cell lines, BGN has been linked to the promotion of angiogenesis through the overexpression of VEGF [36]. OGN is the other molecular species whose expression was altered in both RS- and LSCCs although, contrary to BGN, it underwent a downregulation. Again, this finding agrees with previously described results in different types of tumors such as gastric [37], breast [38], squamous cervical and vaginal [39] and laryngeal [40]. The deregulation of OGN has also been observed in colorectal adenomas, the major precursor lesion of colorectal cancer [41], the overexpression of this gene in mouse hepatocellular carcinoma caused a decrease in invasion and metastasis capacity [41], while its overexpression in CCs has been related to longer survival and the restriction of tumor progression [42].

In LSCCs, three significant expression alterations that were not detectable in RSCCs were observed specifically PRELP, CHAD and PODN, all of which underwent downregulation in the tumor tissues. An important finding since previous information on the role of these molecules in the development of tumors is limited. However, proteomic analysis of CC structures, readily accessible form the tumor vasculature, has described the existence of a reduction in PRELP expression [43]. In the case of CHAD, its role as a tumor repressor has been described, with reduced levels of expression in hepatocarcinomas that was associated with poor survival and an increased tendency to metastasis. In addition, the reduction of CHAD expression in tumor cell lines produced an increase in their capacity for migration and proliferation [44]. Finally, PODN is a molecule that shows antimigratory and antiproliferative properties [45] and has been shown to be a potent inhibitor of migration and proliferation when overexpressed in smooth muscle cells [46], while silencing its coding gene produces increased cell proliferation [47].

The small size of the population sample in this work limits the survival analysis. However, our series is consistent with the results from other large prospective studies regarding survival and the clinical characteristics of the tumor. Better RFS was also observed in cases of RSCC, and, in line with previous studies, it was observed that after complete resection these tumors have better prognosis, although this behavior is modified once the tumor recurs, when it becomes more aggressive and is less responsive to chemotherapy. Additionally, of the SLRPs that showed alterations in their expression pattern, statistical significance was observed only in the case of PODN, where its underexpression was linked with better RFS, which suggests its potential as a prognostic factor. The finding that PODN subexpression was only associated to a statistically significant extent with RFS may be due to the fact that this survival measure is the one with the least heterogeneity among patients. After tumor recurrence, various different therapies could be recommended for different patients. Each involves its own complications, and each patient may respond differently, all of which potentially impacts on the other two survival measures (PFS and OS), complicating the possibility of finding associations between them and alterations in SLRPs. The underexpression of OGN, CHAD and PRELP was also linked to better survival, while overexpression of BGN was linked to worse outcomes. OGN underexpression, specifically, was related to better outcomes in terms of event free survival, relapse or progression, indicating its possible utility as a marker of disease aggressiveness regardless of the stage of the tumor at diagnosis. Prospective studies are needed to elucidate the prognostic role of these molecules.

Given the lack of cell lines typical of RSCCs and LSCCs, we studied the possible involvement of SLRPs in CCs using the human colon adenocarcinoma cell line HT29. The study was carried out using the two molecules whose expression was altered in CCs regardless of their anatomical location, BGN and OGN, as well as PRELP, which appeared underexpressed only in LSCCs. Given that these molecules are all located in the ECM, the tests were carried out growing the cells on plates coated with collagen and one of the molecules analyzed. The motility tests demonstrated no statistically significant differences between the samples. However, the presence of the any of the three SLRP analyzed on the surface of the culture plates was found to reduce cell proliferation, which was especially intense in the case of BGN. It is, however, difficult to extrapolate these results to those obtained in patients because, as indicated above, there is a certain degree of functional overlap between the SLRPs that leads to the existence of compensatory mechanisms between them [23], while in these tests individual molecules were analyzed. Furthermore, it is known that the complex structure and composition of the ECM plays a role in the progression of cancer, with many of its components appearing dysregulated during tumor development [8,9]. Finally, the results presented in this work showed an aberrant intracellular localization of some SLRPs, which would influence the normal performance of their functions, including interactions between cells and the ECM and the regulation of cell signalling [10]. Despite these limitations, it is of interest to highlight the effect that the molecules analyzed showed in terms of reducing cell proliferation, which could be related to the influence of the subexpressions observed in the samples of patients with greater tumor proliferation. However, levels of BGN were increased in tumors regardless of their location, although this increase was, at least at the RNA level, quantitatively lower than the observed subexpressions, and could be related to the compensatory mechanisms indicated above. In addition, this increase could be related to other types of mechanisms that favor tumor progression, such as the previously described effects of antiapoptotic or pro-angiogenic drugs [34,35].

## 5. Conclusions

In conclusion, this work shows that the transcription of SLRPs in non-neoplastic mucosa from ascending and descending colon showed very similar patterns between the two anatomical locations. However, when analysing the levels of mRNA in the CCs, it was possible to determine a series of alterations that were different depending on whether they were RS- or LSCCs, with a greater number of deregulations occurring in the latter. The alterations included both over- and underexpressions, which could point to the existence of a compensatory effect between the different species of SLRPs. The analysis of protein expression showed comparable results, but also indicated changes in the location of these molecules, which largely moved from the ECM to the cell interior. The in vitro studies using the HT29 tumor cell line suggest that deregulation of these molecules could affect cell proliferation. Taken together, these results suggest that SLRPs constitute a new set of markers to add to the differences already observed in numerous studies between the different types of CCs based on their anatomical location.

## Figures and Tables

**Table 1:** Summary of the clinical data of the patients.

		Primary Tumor Location (PTL)						Total	
		RS-CC			LS-CC				
		*N*	% Total	% RS-CC	*N*	% Total	% LS-CC	*N*	%
Gender	Male	12	48.00	100.00	13	52.00	65.00	25	78.13
	Female	0	0.00	0.00	7	100.00	35.00	7	21.88
Age	≤70 years old	5	27.78	41.67	13	72.22	65.00	18	56.25
	>70 years old	7	50.00	58.33	7	50.00	35.00	14	43.75
pT	pT3	11	34.38	91.67	20	62.50	100.00	31	96.88
	pT2	1	3.12	8.33	0	0.00	0.00	1	3.12
pN	pN0	4	28.57	33.33	10	71.43	50.00	14	43.75
	pN+	8	44.44	66.67	10	55.56	50.00	18	56.25
Grade	Low-grade	8	29.63	66.67	19	70.37	95.00	27	84.38
	High-grade	4	80.00	33.33	1	20.00	5.00	5	15.63
Mucinous	No	11	36.67	91.67	19	63.33	95.00	30	93.75
	Yes	1	50.00	8.33	1	50.00	5.00	2	6.25
Perineural invasion	No	11	37.93	91.67	18	62.07	90.00	29	90.63
	Yes	1	33.33	8.33	2	66.67	10.00	3	9.38
Lymphovascular invasion	No	9	33.33	75.00	18	66.67	90.00	27	84.38
	Yes	3	60.00	25.00	2	40.00	10.00	5	15.63
Ulceration	No	4	26.67	33.33	11	73.33	55.00	15	46.88
	Yes	8	47.06	66.67	9	52.94	45.00	17	53.13
Perforation	No	11	39.29	91.67	17	60.71	85.00	28	87.50
	Yes	1	25.00	8.33	3	75.00	15.00	4	12.50
Inflammatory infiltrate	No	1	14.29	50.00	6	85.71	54.55	7	53.85
	Yes	1	16.67	50.00	5	83.33	45.45	6	46.15
Metastatic at diagnosis	No	8	36.36	66.67	14	63.64	70.00	22	68.75
	Yes	4	40.00	33.33	6	60.00	30.00	10	31.25
Metastatic any time	No	5	31.25	41.67	11	68.75	55.00	16	50.00
	Yes	7	43.75	58.33	9	56.25	45.00	16	50.00
CEA at diagnosis	Normal	4	26.67	36.36	11	73.33	68.75	15	55.56
	High	7	58.33	63.64	5	41.67	31.25	12	44.44

## Data Availability

The data presented in this study are contained within the article or Supplementary Materials.

## Supplementary Materials

The following are available online at https://www.mdpi.com/article/10.3390/cells10082002/s1, Table S1: qRT-PCR primer sequences, Table S2: Relationship between alteration in gene expression and survival data, Univariate analysis for RFS and PFS, Figure S1: Relapse Free Survival in SLRPs with differences in their expression pattern. Figure S2: Progression Free Survival in SLRPs with differences in their expression pattern.
