# Supplementary material for: The Genes Encoding Small Leucine-Rich Proteoglycans Undergo Differential Expression Alterations in Colorectal Cancer, Depending on Tumor Location
Source: Cells. 2021 Aug 6;10(8):2002. doi: 10.3390/cells10082002 (PMC8391422; doi:10.3390/cells10082002)
Supplement: Supplementary file 1 [file cells-10-02002-s001.zip › Supplementary figure legends.pdf]

Supplementary figure legends:

Figure S1: Relapse Free Survival in SLRPs with differences in their expression pattern. (**A**) Kaplan-Meier curves for Relapse Free Survival. Only PODN showed statistically significative differences. (**B**) Forest Plot for Cox Regression model for SRLPs displaying alterations. PODN appears as a protection factor when underexpressed.

Figure S2: Progression Free Survival in SLRPs with differences in their expression pattern. (**A**) Kaplan-Meier curves for Progression Free Survival. Only PODN showed statistically significative differences. (**B**) Forest Plot for Cox Regression model displaying no statistically differences for the SLRPs in PFS.
