# Supplementary material for: The Genes Encoding Small Leucine-Rich Proteoglycans Undergo Differential Expression Alterations in Colorectal Cancer, Depending on Tumor Location
Source: Cells. 2021 Aug 6;10(8):2002. doi: 10.3390/cells10082002 (PMC8391422; doi:10.3390/cells10082002)
Supplement: Supplementary file 1 [file cells-10-02002-s001.zip › Supplementary table S1.pdf]

Supplementary table S1

qRT-PCR primer sequences

Gene	Gene ID	Primer sequence
LUM	4060	F 5'- TCACATGCCACACCACAAGA<br>R 5'- TGAGTGATACAAGAGCTGAAGGG
DCN	1634	F 5'- GGCTTCTTATTCGGGTGTGAGTC<br>R 5'- AGTTTCCGAGTTGAATGGCAGAG
KERA	11081	F 5'- GGTTCCCCGAATCAGTGCTC<br>R 5'- CGGAGGTAGCGAAGATGAGG
BGN	633	F 5'- GGTCTGAAGTCTGTGCCCAAA<br>R 5'- GAGGTGCTGGAGACCCTTGA
OGN	4969	F 5'- CCACAGTCAGCAGGCCACTA<br>R 5'- GATAATGCGTGAGTCCTGCTG
ASPN	54829	F 5'- CTCAGTCCCAACCAACATTCCA<br>R 5'- TGTTGTTGTTCAGGATCAGACCA
ECM2	1842	F 5'- CAAGTCTGGAGCTCACTGGC<br>R 5'- ATGCTTTTGGACCTATGCCTGA
FMOD	2331	F 5'- AGCAGCCTCCTTGAGCTAGA<br>R 5'- AAGTTCACGACGTCCACCAC
PRELP	5549	F 5'- GCCATCAACAACAGGCTGGA<br>R 5'- CCAGGTCCGAGGAGAAGTCA
EPYC	1833	F 5'- GGGGCCACACACAAATGAAGA<br>R 5'- TTTGGCAGCGGAGGAATAGC
OPTC	26254	F 5'- CCCCACGATGACCAGACCTA<br>R 5'- TACACAGAGGAACCGAGGCA
TSK	25987	F 5'- GACAACCCGGCCATGCTTC<br>R 5'- CGCTACAATCCACCCGAGTC
CHAD	1101	F 5'- CAAGTTCAAGGGCCAGCACA<br>R 5'- TAGTCTCTCCGGTGGAAGGC
NYX	60506	F 5'- GTGACCTGTCCTTTCTCCCCT<br>R 5'- GGACCACCGCATGCAGAAG
PODN	127435	F 5'- CCCGAGTCACTTGAGTACCTGTA<br>R 5'- AGCCAGCTTGTTAAACCTGAGAA
PODNL1	79883	F 5'- TCGAGTGTTCCCGGACAACA<br>R 5'- GGGACAGCTCATTGTAGGGGA
OMD	4958	F 5'- CCCCTGAAAACTAAAGCAAATTGAACA<br>R 5'- CTGGCTCTTGGTCATAGTCTTCA

The genes encoding small leucine-rich proteoglycans undergo differential expression alterations in colorectal cancer, depending on tumor location

International Journal of Colorectal Disease

M.P. Solis-Hernandez, B. García, N. Pérez-López, Y. García-Mesa, S. González-Fernández, O. García-Suárez, J. Merayo, C. Martín, I. Fernández-Vega, L.M. Quirós

Luis M. Quirós. Department of Functional Biology, University of Oviedo, Oviedo, Spain. Email: quirosluis@uniovi.es

Iván Fernández-Vega. Department of Surgery and Medical-surgical Specialties, University of Oviedo, Oviedo, Spain Email: fernandezvivan@uniovi.es
