# Supplementary material for: The Genes Encoding Small Leucine-Rich Proteoglycans Undergo Differential Expression Alterations in Colorectal Cancer, Depending on Tumor Location
Source: Cells. 2021 Aug 6;10(8):2002. doi: 10.3390/cells10082002 (PMC8391422; doi:10.3390/cells10082002)
Supplement: Supplementary file 1 [file cells-10-02002-s001.zip › Supplementary table S2.pdf]

Supplementary table S2

Relationship between alteration in gene expression and survival data

Univariate analysis for RFS and PFS

		RFS					PFS				
		n	Events	Median *months*	p	HR (95%CI)	n	Events	Median *months*	p	HR (95%CI)
	**All Patients**	**25**	**14**	**27.30**			**32**	**21**	**21.87**		
**PTL**	RSCC*	7	3	40.03*	0.953	0.962 (0.267-3.464)	12	7	17.20	0.669	1.104 (0.701-1.740)
	LSCC§	18	11	21.43			20	14	19.47*		
**Age**	<65 years old	9	7	14.20	***0.072***	0.375 (0.129-1.091)	12	10	14.20	0.281	0.623 (0.262-1.483)
	≥65 years old *§	16	7	40.30			20	11	27.30		
**BGN**	No overexpression*§	8	3	NR	0.207	1,514 (0,795-2,882)	11	6	21,53	0.560	1,153 (0,714-1,862)
	Overexpression	17	11	16.93			21	15	21,87		
**OGN**	No underexpression	10	7	16.93	0.352	0,606 (0,211-1,739)	13	10	21,53	0,771	0,877 (0,363-2,119)
	Underexpression*§	15	7	40.03			19	11	21,87		
**CHAD** *n=17*	No underexpression	2	1	14.20	0.763	1,372 (0,175-10,757)	4	3	12,00	0.549	0,672 (0,183-2,464)
	Underexpression*§	15	8	21.43			16	11	21,53		
**PRELP** *n=17*	No underexpression*§	5	3	19.47	0.950	0,958 (0,254-3,619)	6	4	12,00	0.959	0,970 (0,303-3,106)
	Underexpression	12	8	21.43			14	10	21,43		
**PODN** *n=17*	No underexpression	6	5	12.23	***0.45***	***0.289 (0.086-0.975)***	7	6	12,00	0.246	0,533 (0,1840-1,545)
	Underexpression*§	11	6	27.30			13	8	21,53		

n=25 patients after R0 resection (complete resection of primary ± metastasis) at diagnosis.
NR: not reached
** indicates better RFS*
§ indicates better PFS
