# Supplementary figures and images for: The Genes Encoding Small Leucine-Rich Proteoglycans Undergo Differential Expression Alterations in Colorectal Cancer, Depending on Tumor Location
Source: Cells. 2021 Aug 6;10(8):2002. doi: 10.3390/cells10082002 (PMC8391422; doi:10.3390/cells10082002)

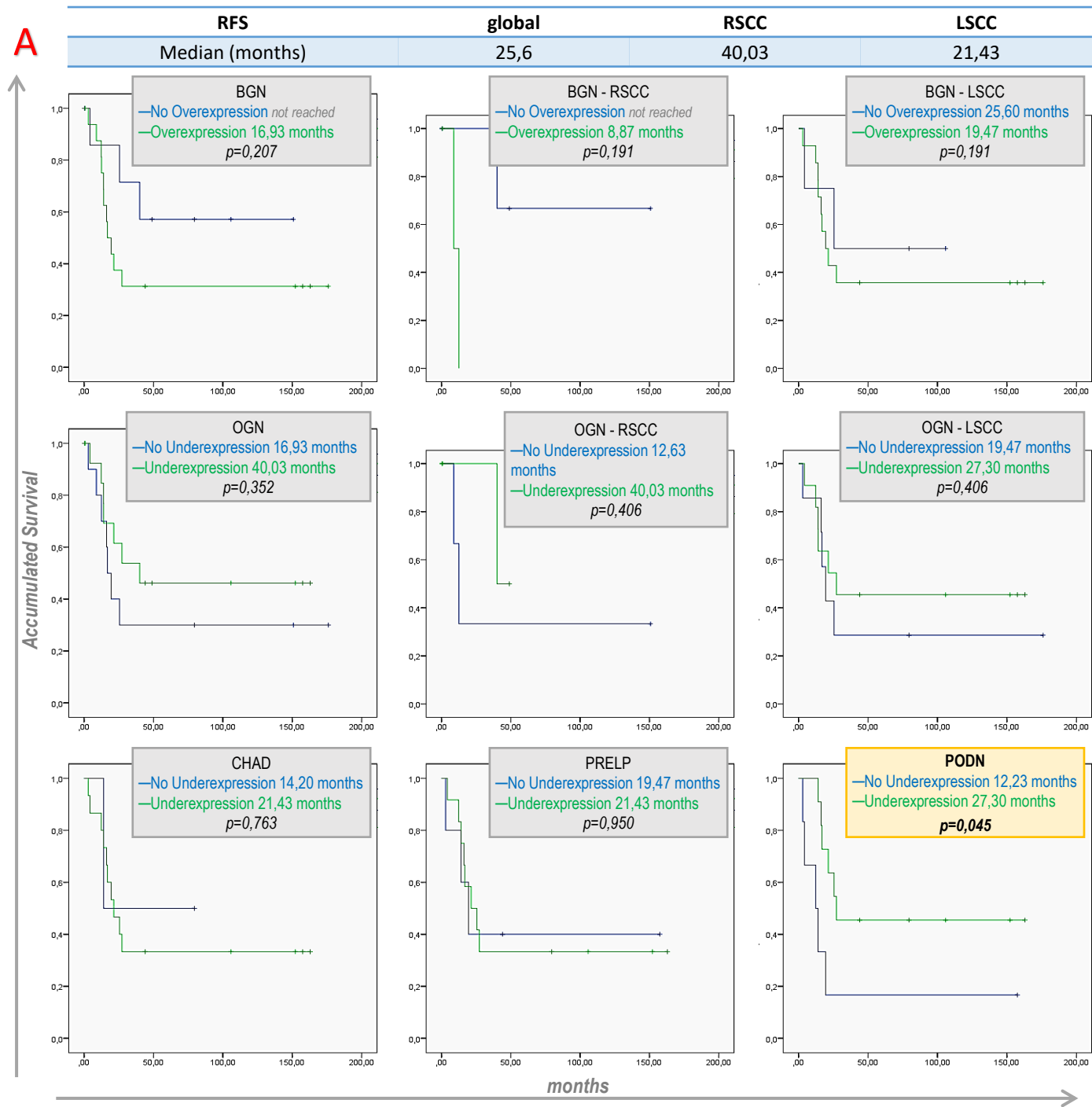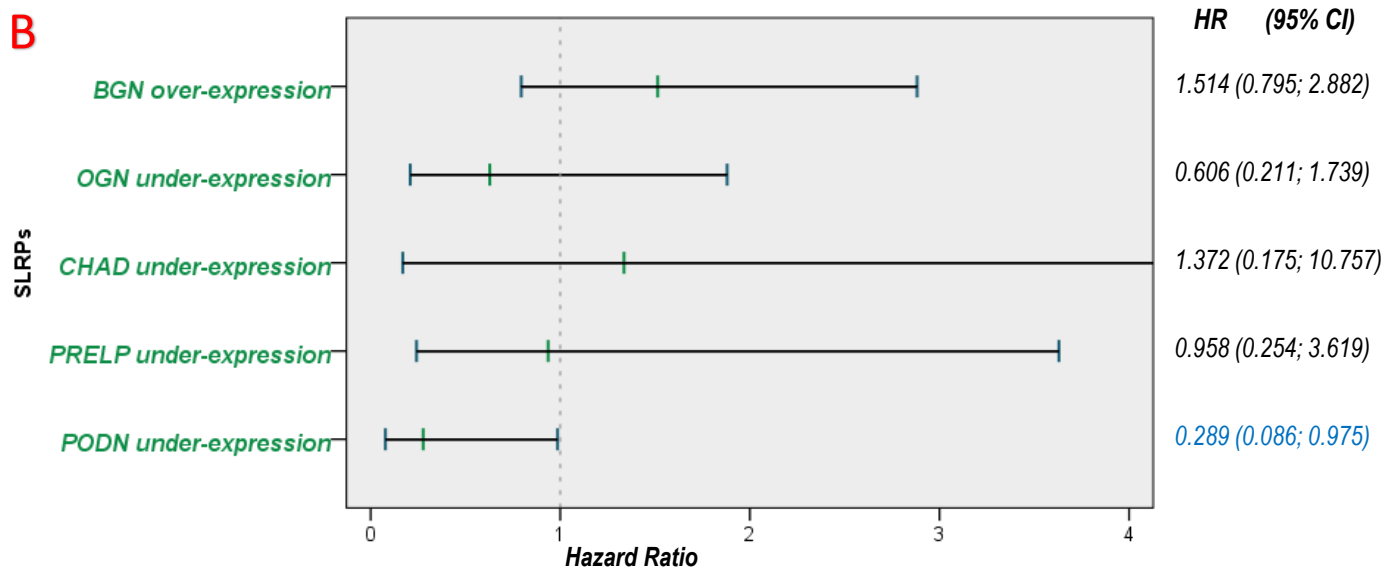

Supplement: Supplementary file 1 [file cells-10-02002-s001.zip › Supplementary figure S1.pdf]

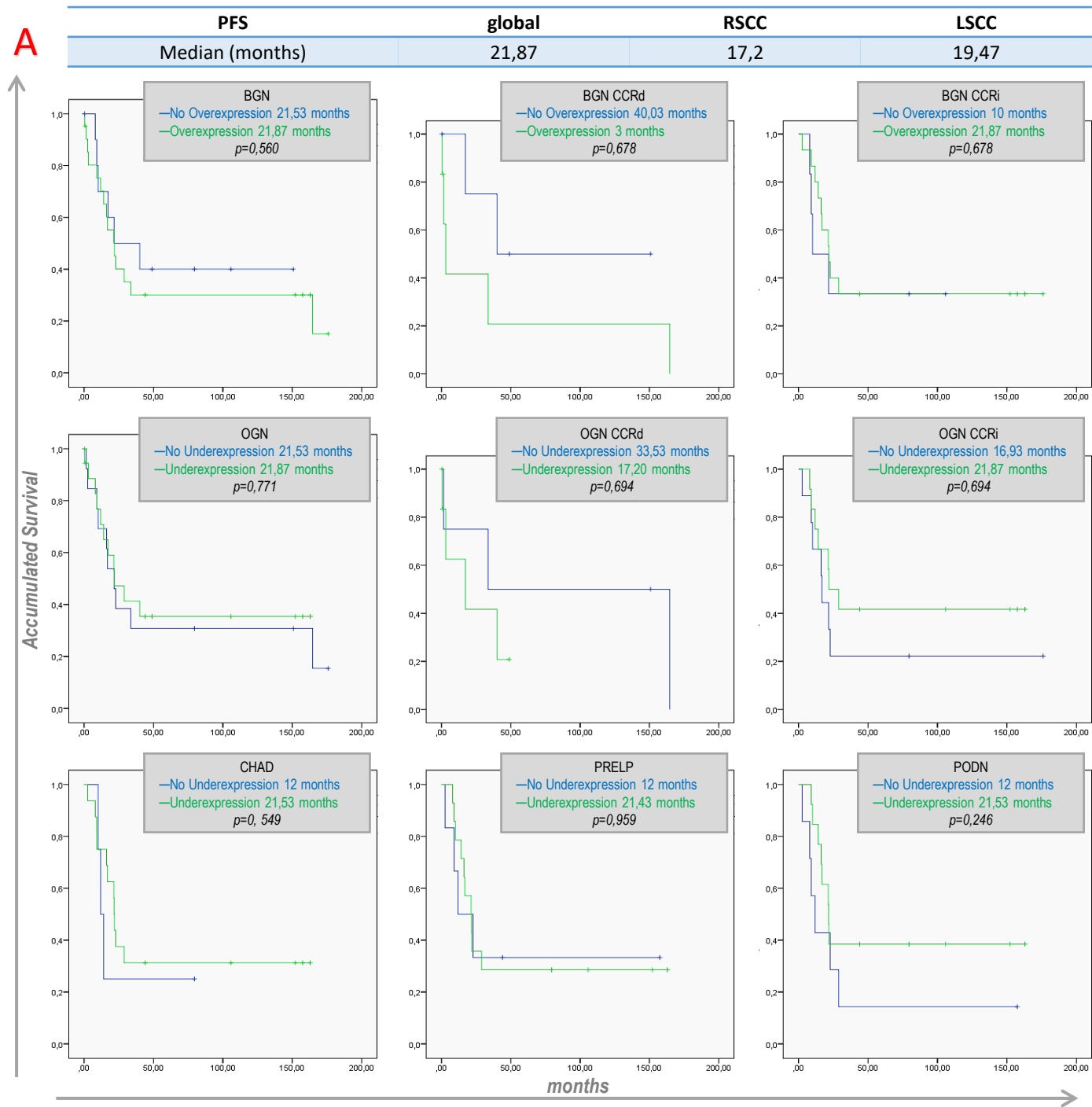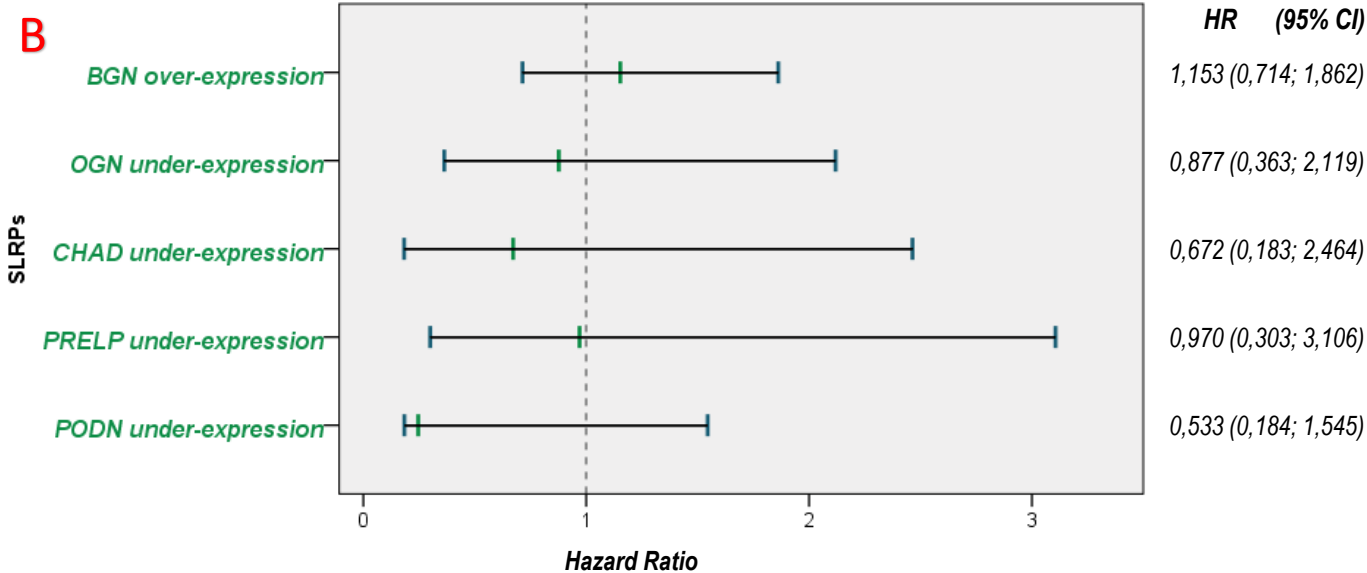

Supplement: Supplementary file 1 [file cells-10-02002-s001.zip › Supplementary figure S2.pdf]
